# Supplementary figures and images for: Toxicity of Melaleuca alternifolia essential oil to the mitochondrion and NAD+/NADH dehydrogenase in Tribolium confusum
Source: PeerJ. 2018 Nov 13;6:e5693. doi: 10.7717/peerj.5693 (PMC6238770; doi:10.7717/peerj.5693)

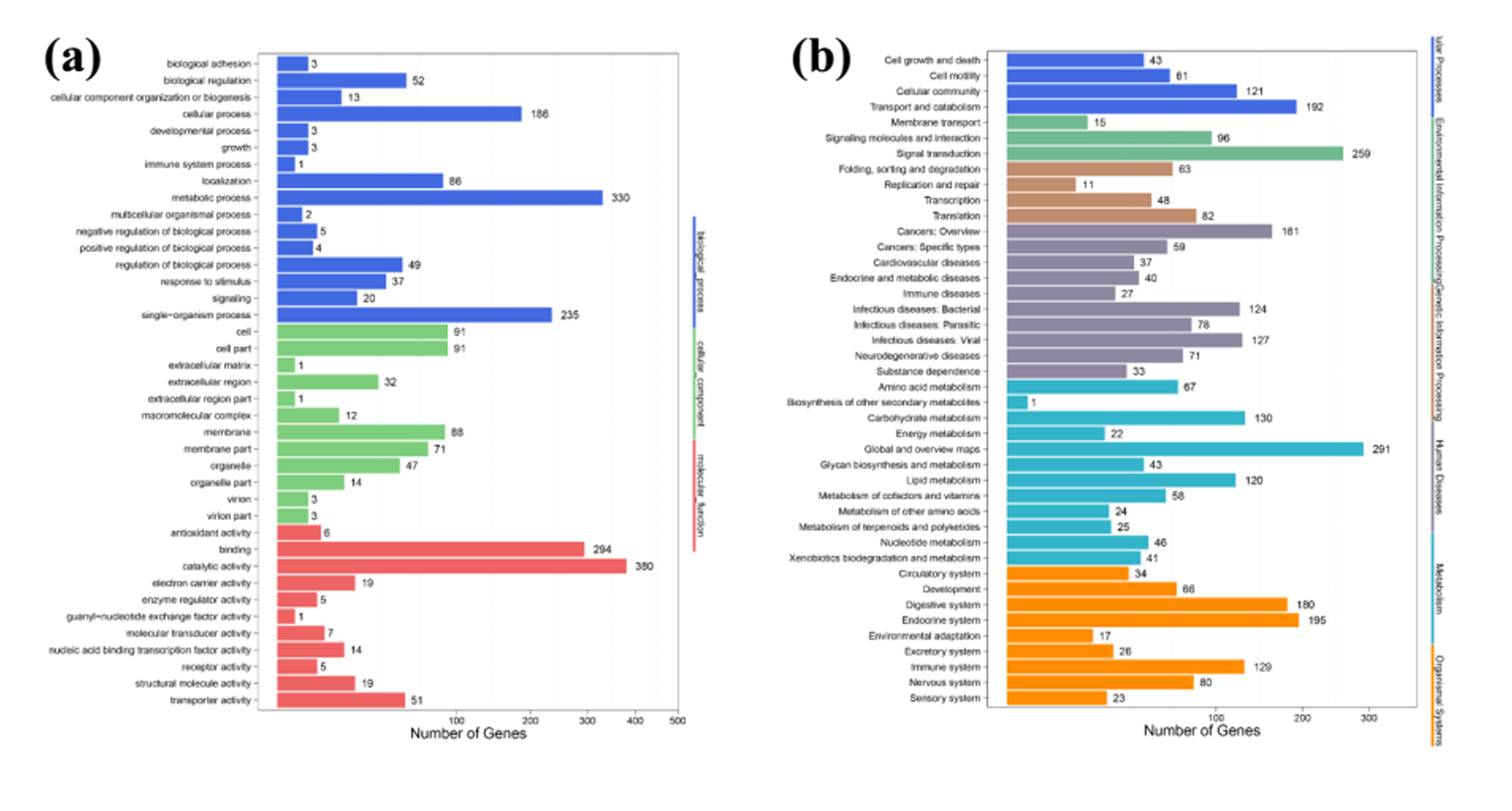

Supplement: Supplemental Information 1 [file peerj-06-5693-s001.jpg]
