# Supplementary material for: Toxicity of Melaleuca alternifolia essential oil to the mitochondrion and NAD+/NADH dehydrogenase in Tribolium confusum
Source: PeerJ. 2018 Nov 13;6:e5693. doi: 10.7717/peerj.5693 (PMC6238770; doi:10.7717/peerj.5693)
Supplement: Supplemental Information 7 [file peerj-06-5693-s007.pdf]

INSufhE [Transcriptome Denovo Project]

BGI Co., Ltd.

Wednesday, 2nd Mar., 2016

Table of Contents

Results 2
- 1 Abstract 2
- 2 Sequencing Reads Filtering 2
- 3 De novo Assembly 3
- 4 Unigene Functional Annotation 3
- 5 Unigene CDS Prediction 6
- 6 Unigene SSR Detection 7
- 7 SNP Detection 9
- 8 Unigene Expression 10
- 9 DEG Detection 10
- 10 Gene Ontology Analysis of DEG 11
- 11 Pathway Analysis of DEG 12

Methods 13
- 1 Transcriptome De novo Study Process 13
- 2 Sequencing Reads Filtering 14
- 3 De novo Assembly 14
- 4 Unigene Functional Annotation 15
- 5 Unigene CDS Prediction 16
- 6 Unigene SSR Detection 16
- 7 SNP Detection 16
- 8 Unigene Expression 17
- 9 DEG Detection 17
- 10 Gene Ontology Analysis of DEG 17
- 11 Pathway Analysis of DEG 17

Help 17
- 1 FASTQ Format 17
- 2 Annotation list format 18
- 3 What is TF 19
- 4 Gene expression list format 19
- 5 DEG list format 19
- 6 MA plot 19
- 7 Volcano plot 20
- 8 Cluster list format 20
- 9 VCF format 20
- 10 How to read DEG GO enrichment analysis result 20
- 11 How to read DEG pathway enrichment analysis result 20

References 21

Results

1 Abstract

In our project, we generated about 8.93 Gb bases in total after Illumina Hiseq sequencing. Then assemble all samples together, we got 28,885 Unigenes, the total length, average length, N50, and GC content of Unigenes are 36,998,010 bp, 1,280 bp, 2,097 bp, and 37.39 %, respectively. And then annotate Unigenes with 7 functional databases, finally, 23,160(NR: 80.18%), 9,941(NT: 34.42%), 18,187(Swissprot: 62.96%), 9,451(COG: 32.72%), 18,074(KEGG: 62.57%), 6,333(GO: 21.92%), and 17,837(Interpro: 61.75%) Unigenes are annotated. With functional annotation results, we detected 23,102 ***CDS***, and after predected by ESTScan with the remaining Unigenes, we got 735 ***CDS*** more. We also detected 1,683 ***SSR*** distribute on 1,501 Unigenes.

2 Sequencing Reads Filtering

The sequencing reads which containing low-quality, adaptor-polluted and high content of unknown base(N) reads, should be processed to remove this reads before downstream analyses. After filtering, reads quality metrics are shown as **Table 1** . The distribution of base content and quality are shown as **Figure 1** and **Figure 2** , respectively.

Table 1 Summary of sequencing reads after filtering. (Download)

Sample	Total Raw Reads(Mb)	Total Clean Reads(Mb)	Total Clean Bases(Gb)	Clean Reads Q20(%)	Clean Reads Q30(%)	Clean Reads Ratio(%)
ch-ck	64.76	45.11	4.51	97.38	93.87	69.66
ch-cr	61.52	44.23	4.42	97.55	94.19	71.89

Q20: the rate of bases which quality is greater than 20.

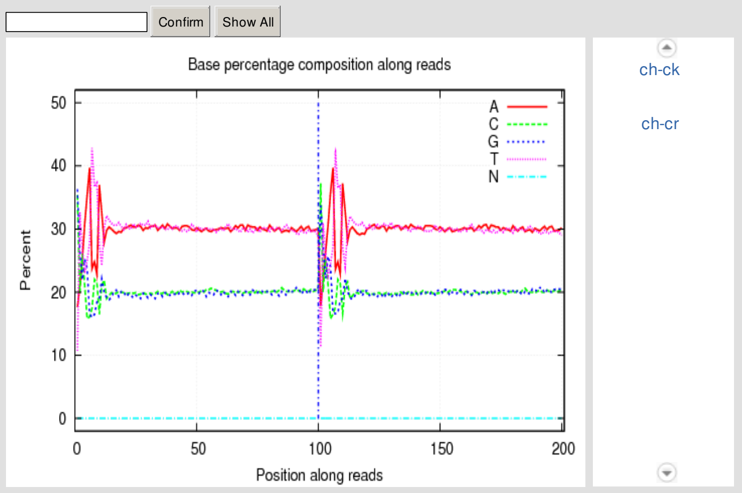

Figure 1 Distribution of base composition on clean reads. X axis represents base position along reads.Y axis represents base content percentage. As to high quality sequencing reads, A(adenine base) curve should be strictly overlapped with T(thymine base) curve and G(guanine bsase) curve should be overlapped with C(cytosine base) curve according to the principle of complementary of base pairing, excluding the first six base positions owing to Illumina sequencing platform using random hexamer-primer to synthesize cDNA which could result in PCR bias. As shown if figure, big fluctuations in first six base positons along reads, it is normal situation. If abnormal condition happens during sequencing, it may show an unbalanced composition.

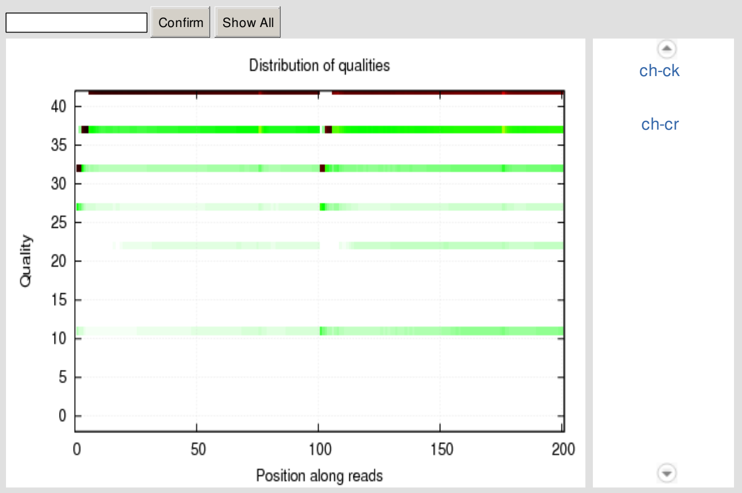

Figure 2 Distribution of base quality on clean reads. X axis represents base positions along reads. Y axis represents base quality value. Each dot in the image represents the number of total bases with certain quality value of the corresponding base along reads. Darker dot color means greater bases number. If the percentage of the bases with low quality

(< 20) is very high, then the sequencing quality of this lane is bad.

3 De novo Assembly

After reads filtering, we use Trinity [1] to perform de novo assembly with clean reads, the assembly quality metrics are shown as **Table 2** , and the transcript length distribution is shown as **Figure 3** . Next step, we use Tgicl [2] to cluster transcripts to Unigenes, the clustering quality metrics are shown as **Table 3** , and the Unigene length distribution is shown as **Figure 4** .(if more than one sample, we would exexecute Tgicl again with each sample's Unigene to get final Unigene for downstream analyses, named as "All-Unigene")

Table 2 Quality metrics of transcripts (Download)

Sample	Total Number	Total Length	Mean Length	N50	N70	N90	GC(%)
ch-ck	34,747	36,246,577	1,043	1,882	1,187	422	37.39
ch-cr	33,988	35,442,812	1,042	1,870	1,188	425	37.30

N50: a weighted median statistic that 50% of the TotalLength is contained in transcripts great than or equal to this value. GC (%): the percentage of G and C bases in all transcripts.

Table 3 Quality metrics of Unigenes (Download)

Sample	Total Number	Total Length	Mean Length	N50	N70	N90	GC(%)
ch-ck	26,367	31,599,198	1,198	1,996	1,322	526	37.49
ch-cr	25,883	30,826,842	1,191	1,965	1,319	527	37.41
All-Unigene	28,885	36,998,010	1,280	2,097	1,424	590	37.39

N50: a weighted median statistic that 50% of the TotalLength is contained in Unigenes great than or equal to this value. GC (%): the percentage of G and C bases in all Unigenes.

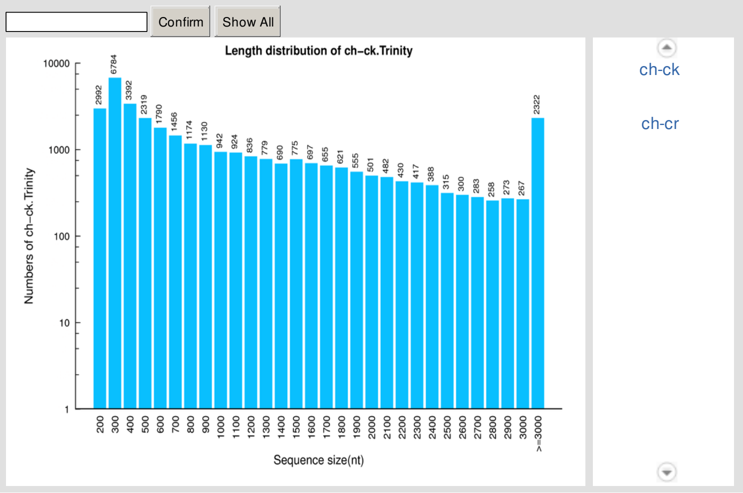

Figure 3 Transcript length distribution. X axis represents the length of transcripts. Y axis represents the number of transcripts.

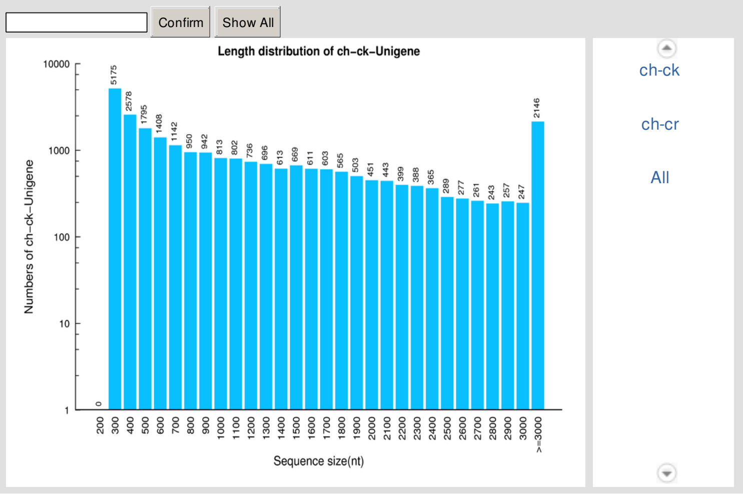

Figure 4 Unigene length distribution. X axis represents the length of Unigenes. Y axis represents the number of Unigenes.

4 Unigene Functional Annotation

After assembly, we perform functional annotation with 7 functional databases(NR, NT, GO, COG, KEGG, Swissprot and Interpro) for Unigenes, the annotation summary is shown as **Table 4** . With NR annotation, the distribution of annotated species is statisticed, shown as **Figure 5** . And with COG, GO, KEGG annotation, function distribution is statisticed, shown as **Figure 6** , **Figure 7** and **Figure 8** , respectively. We also display annotated Unigenes between NR, COG, KEGG, Swissprot and Interpro with venn diagram, shown as **Figure 9** .

Table 4 Summary of functional annotation result (Download)

Values	Total	Nr-Annotated	Nt-Annotated	Swissprot-Annotated	KEGG-Annotated	COG-Annotated	Interpro-Annotated	GO-Annotated	Overall
Number	28,885	23,160	9,941	18,187	18,074	9,451	17,837	6,333	23,571
Percentage	100%	80.18%	34.42%	62.96%	62.57%	32.72%	61.75%	21.92%	81.60%

Overall: the number of Unigenes which be annotated with at least one functional database.

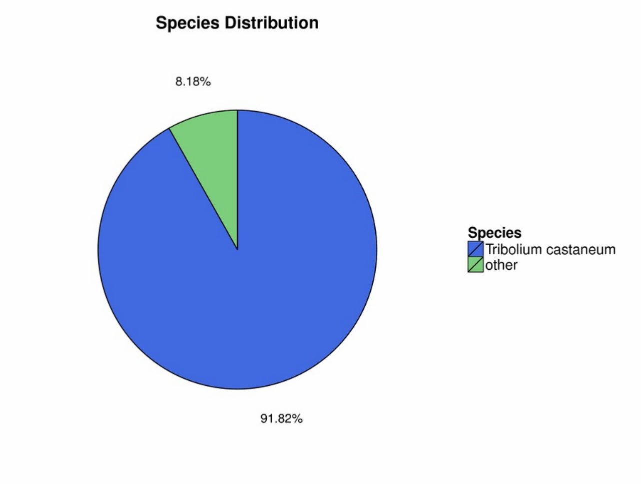

Figure 5 Distribution of annotated species.

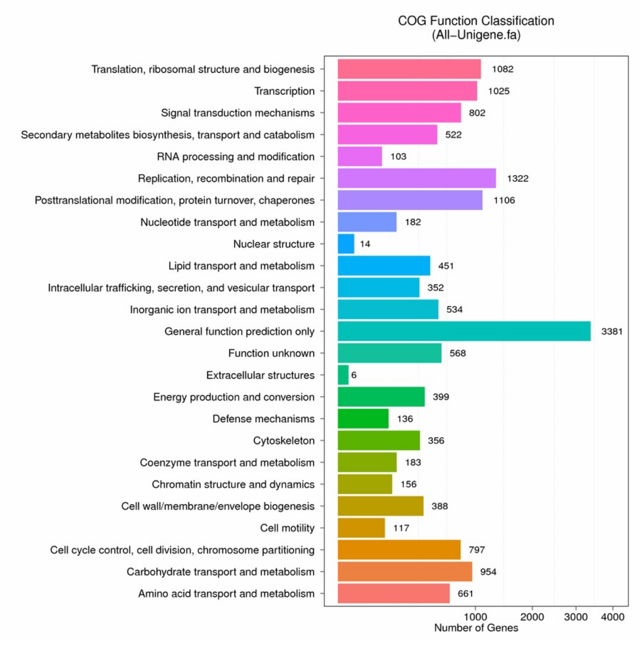

Figure 6 Functional distribution of COG annotation. X axis represents the number of Unigenes. Y axis represents the COG functional category.

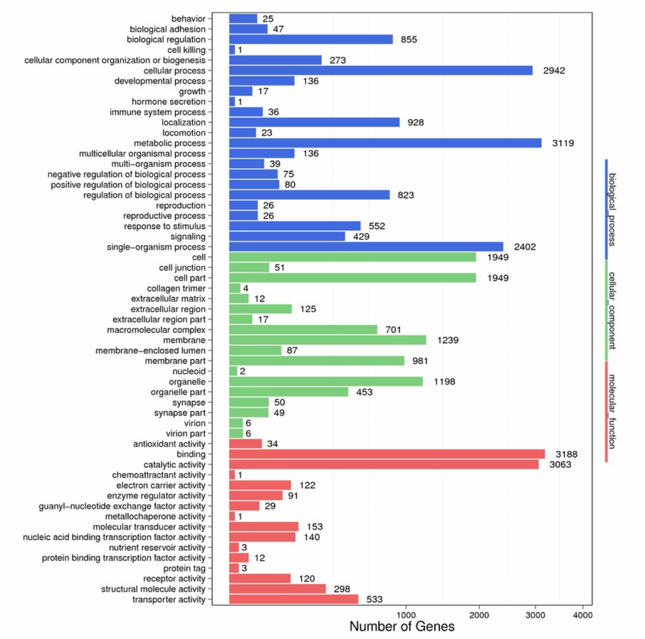

Figure 7 Functional distribution of GO annotation. X axis represents the number of Unigenes. Y axis represents the Gene Ontology functional category.

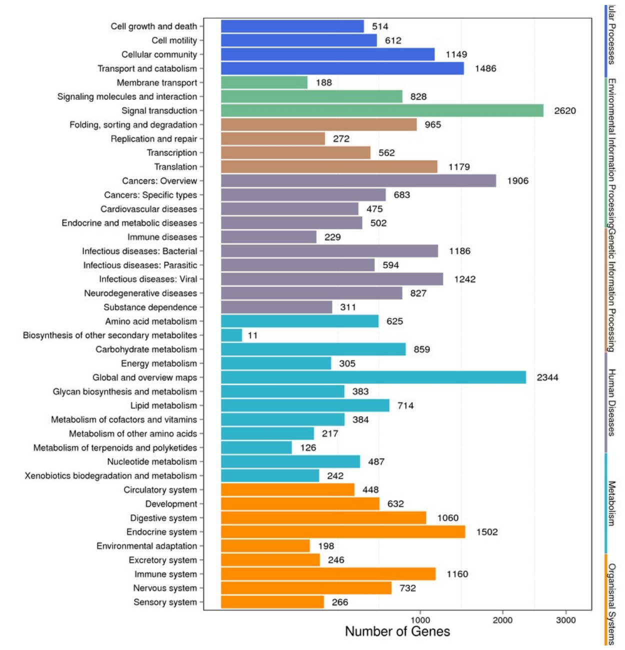

Figure 8 Functional distribution of KEGG annotation. X axis represents the number of Unigenes. Y axis represents the KEGG functional category.

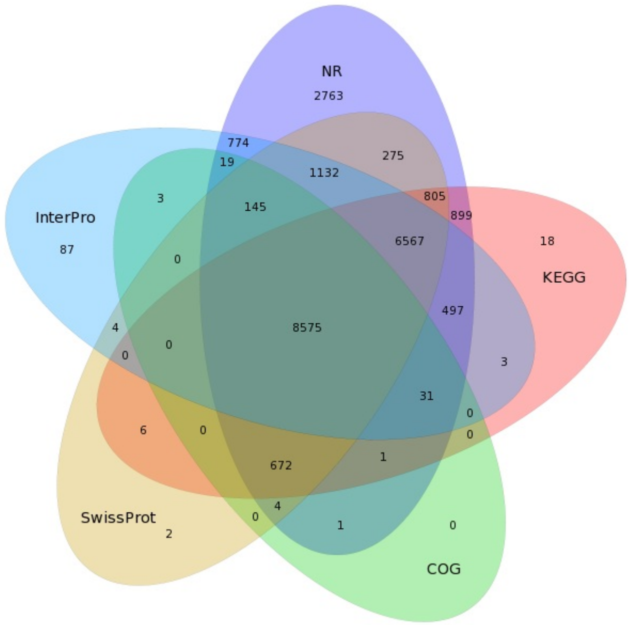

Figure 9 Venn diagram between NR, COG, KEGG, Swissprot and Interpro.

The annotation result of each functional database are shown as tables below, and the overall summarized table is shown as **Table 12** (see Annotation list format in help page).

Table 5 Unigene NR annotation result. (Download)

Table 6 Unigene NT annotation result. (Download)

Table 7 Unigene GO annotation result. (Download)

Table 8 Unigene COG annotation result. (Download)

Table 9 Unigene KEGG annotation result. (Download)

Table 10 Unigene Swissprot annotation result. (Download)

Table 11 Unigene Interpro annotation result. (Download)

Table 12 The overall summarized annotation result. (Download)

5 Unigene CDS Prediction

After functional annotation, we select the segment of Unigene that best mapped to functional databases as its ***CDS***. For the Unigenes that unannotated, we use ESTScan[3] to predict ***CDS***. Prediction summary is shown as **Table 13**, and the distribution of ***CDS*** length is shown as **Figure 10**.

Table 13 Quality metrics of predicted CDS (Download)

Software	Total Number	Total Length	Mean Length	N50	N70	N90	GC(%)
Blast	23,102	24,125,637	1,044	1,548	1,077	510	39.84
ESTScan	735	240,438	327	324	255	213	36.97
Overall	23,837	24,366,075	1,022	1,539	1,062	489	39.81

N50: a weighted median statistic that 50% of the TotalLength is contained in CDS great than or equal to this value. GC (%): the percentage of G and C bases in all CDS.

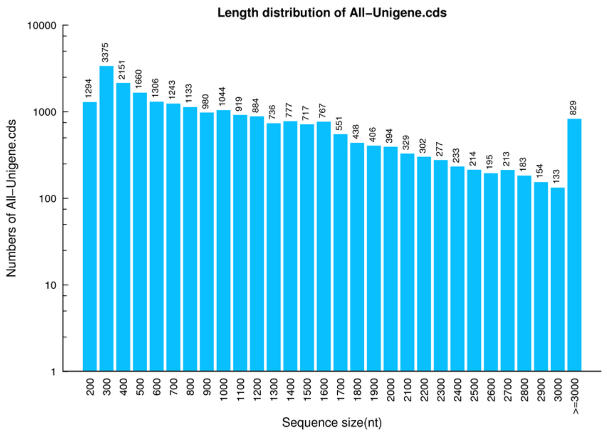

Figure 10 CDS length distribution. X axis represents the length of CDS. Y axis represents the number of CDS.

6 Unigene SSR Detection

After assembly, we detect ***SSR*** in Unigenes, then design primer for each ***SSR*** . ***SSR*** size summary is shown as **Table 14** and **Figure 11** .and the designed primer result is shown as **Table 15** .

Table 14 SSR size summary (Download)

Number	Mono-nucleotide	Di-nucleotide	Tri-nucleotide	Quad-nucleotide	Penta-nucleotide	Hexa-nucleotide
4	0	0	0	0	18	4
5	0	0	503	15	4	0
6	0	97	158	1	0	0
7	0	46	33	0	0	0
8	0	14	21	0	0	0
9	0	7	1	0	1	0
10	0	5	0	0	0	0
11	0	7	2	0	0	0
12	247	2	1	0	0	0
13	117	0	0	0	1	0
14	102	1	1	0	0	0
15	64	0	1	0	0	0
16	62	0	1	0	0	0
17	22	0	0	0	0	0
18	21	1	2	0	0	0
19	6	0	0	0	0	0
20	9	2	0	0	0	0
21	5	3	1	0	0	0
22	1	0	0	0	0	0
23	23	0	0	0	0	0
24	2	0	0	0	0	0
26	3	0	0	0	0	0
28	6	0	0	0	0	0
29	3	0	0	0	0	0
30	2	0	0	0	0	0
31	7	2	0	0	0	0
32	3	1	0	0	0	0
33	2	0	0	0	0	0
35	1	0	0	0	0	0
36	1	0	0	0	0	0
37	1	0	0	0	0	0
38	1	0	0	0	0	0
40	3	0	0	0	0	0
42	1	0	0	0	0	0
43	1	0	0	0	0	0
45	1	0	0	0	0	0
50	1	0	0	0	0	0
51	1	0	0	0	0	0
52	1	0	0	0	0	0
53	1	0	0	0	0	0
55	1	0	0	0	0	0
57	1	0	0	0	0	0
61	1	0	0	0	0	0
62	1	0	0	0	0	0
91	1	0	0	0	0	0
SubTotal	726	188	725	16	24	4

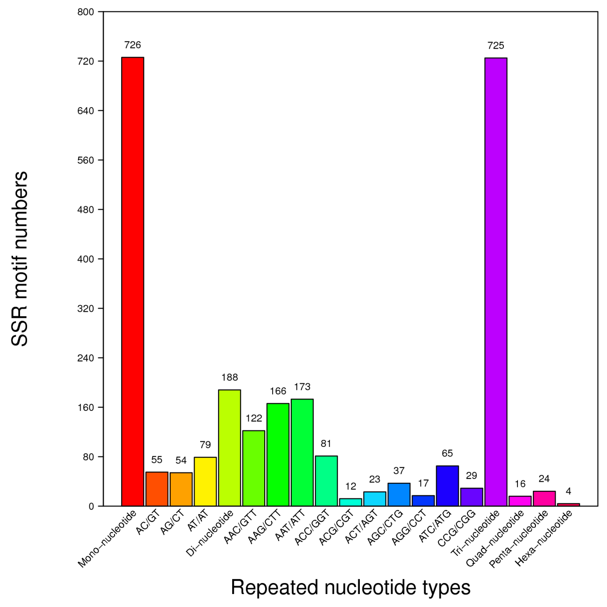

Figure 11 SSR size distribution. X axis represents the type of SSR. Y axis represents the number of SSR.

Table 15 The result of designed primer. (Download)

7 SNP Detection

After assembly, we use GATK[4] to call ***SNP*** variant for each sample with Unigenes as reference. Final results are stored in VCF format. The ***SNP*** summary is shown as **Table 16** , and **Figure 12** . We also generate a friendly-interfaced population ***SNP*** summary in EXCEL format shown as **Table 19** .

Table 16 SNP variant type summary. (Download)

Sample	A-G	C-T	Transition	A-C	A-T	C-G	G-T	Transversion	Total
ch-ck	22,035	22,445	44,480	3,692	6,743	3,279	3,837	17,551	62,031
ch-cr	21,319	21,825	43,144	3,630	6,631	3,208	3,779	17,248	60,392

Transition: variant between purines or pyrimidines.Transversion: variant between purine and pyrimidine.

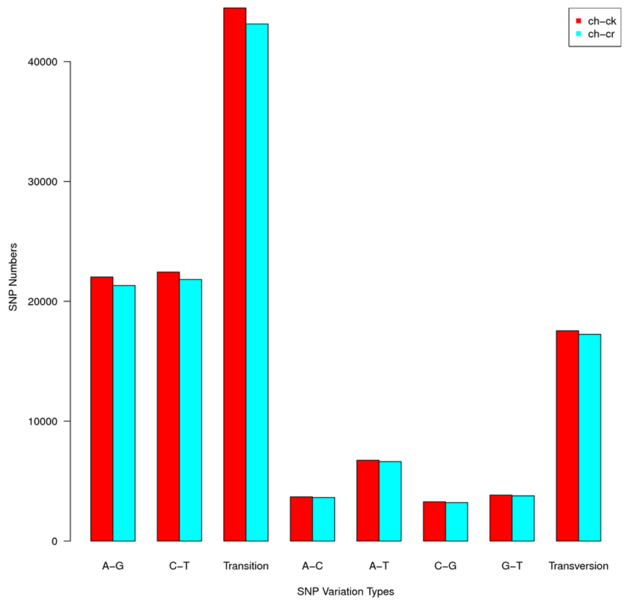

Figure 12 SNP variant type distribution. X axis represents the type of SNP. Y axis represents the number of SNP.

The VCF format ***SNP*** result of each sample are shown as tables below(see VCF format in help page):

Table 17 SNP list of ch-ck (Download)

Table 18 SNP list of ch-cr (Download)

Table 19 Summary of population SNP (Download)

8 Unigene Expression

After assembly, we mapped clean reads to Unigene, then calculate gene expression level for each sample, shown as tables below(see Gene expression list format in help page).

Table 20 Expressed gene list of ch-ck (Download)

Table 21 Expressed gene list of ch-cr (Download)

9 DEG Detection

With Unigene expression result, we detect Differentially Expressed Gene(***DEG***) between samples, the ***DEG*** summary is shown as **Figure 13** .We also show the ***DEG*** distribution using MA plot(see MA plot in help page) and Volcano plot(se Volcano plot in help page), shown as **Figure 14** and **Figure 15** , respectively.

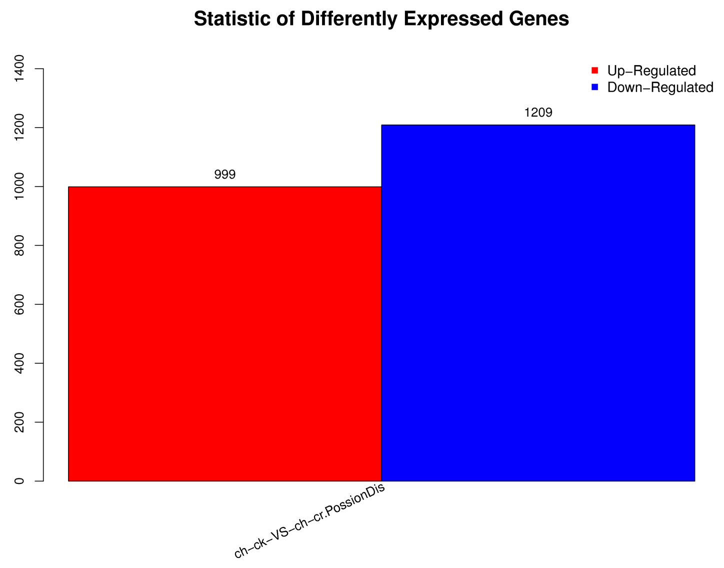

Figure 13 Summary of DEGs. X axis represents comparing samples. Y axis represents DEG numbers. Red color represents up regulated DEGs. Blue color represents down regulated DEGs.

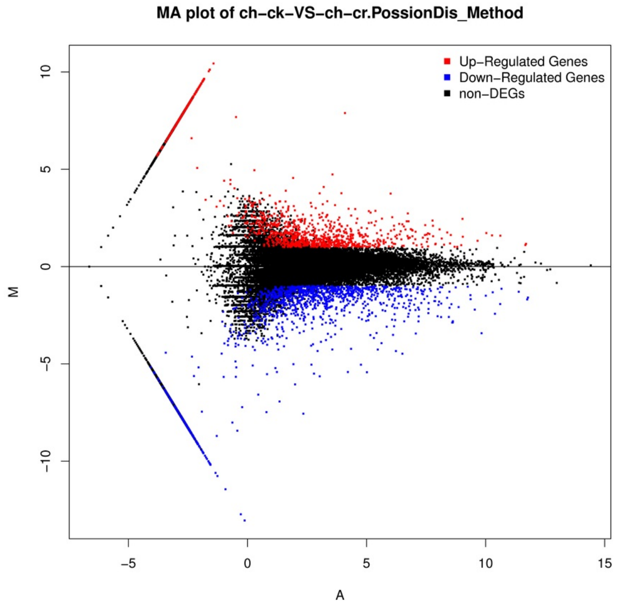

Figure 14 MA plot of DEGs. X axis represents value A(log2 transformed mean expression level). Y axis represents value M(log2 transformed fold change). Red points represent up regulated DEG. Blue points represent down regulated DEG. Black points represent non-DEGs.

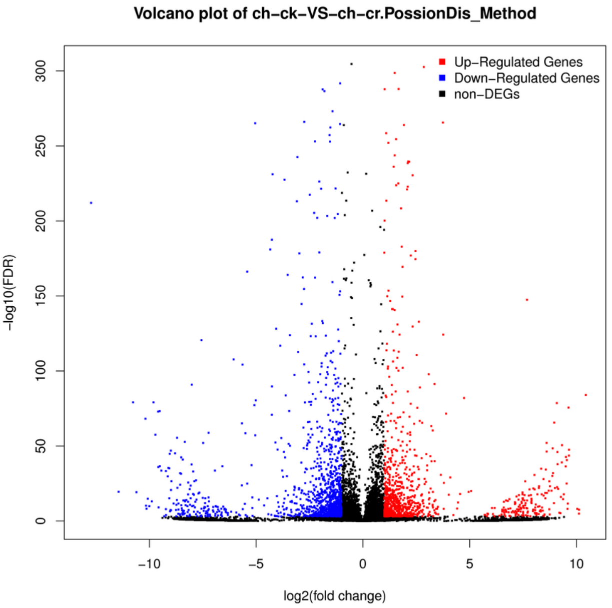

Figure 15 Volcano plot of DEGs. X axis represents -log10 transformed significance. Y axis represents log2 transformed fold change. Red points represent up regulated DEG. Blue points represent down regulated DEG. Black points represent non-DEGs.

DEG lists are shown as tables below(see ***DEG*** list format in help page):

Table 22 DEG list of ch-ck-VS-ch-cr.PossionDis_Method (Download)

10 Gene Ontology Analysis of DEG

With DEGs, we perform ***Gene Ontology*** (GO) classification and functional enrichment for DEGs. GO has three ontologies: molecular function, cellular component and biological process, we would perform functional enrichment respectively. The GO classification results are shown as **Figure 16** , and the GO functional enrichment results are shown as **Figure 17** .

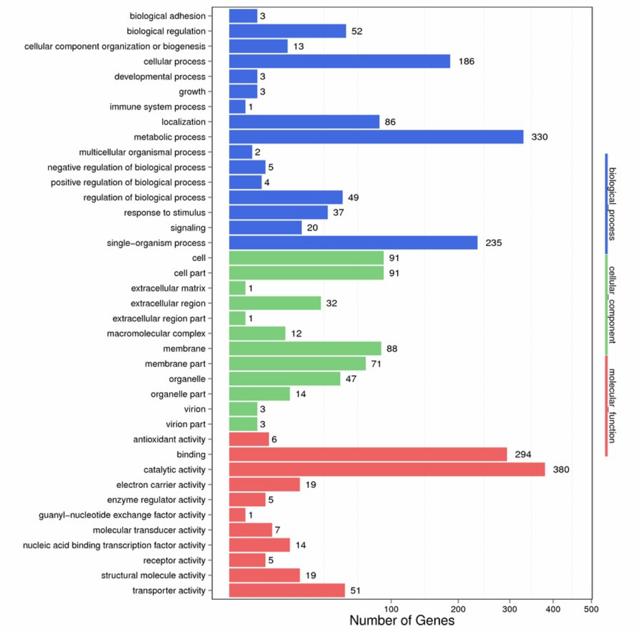

Figure 16 GO classification of DEGs. X axis represents number of DEG. Y axis represents GO term.

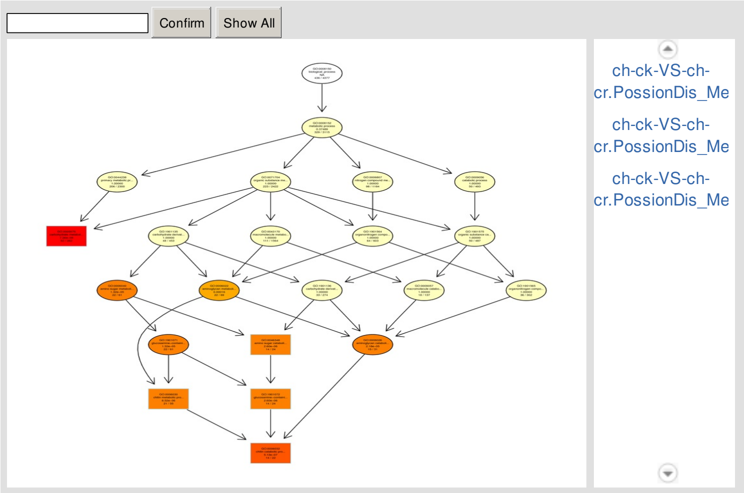

Figure 17 GO functional enrichment of DEGs. Coloring indicate qvalue(high: yellow, low: red). The lower qvalue indicate the more significant enriched.

11 Pathway Analysis of DEG

With DEGs, we perform KEGG pathway classification an functional enrichment for DEGs. The pathway classification results are shown as **Figure 18** , and the pathway functional enrichment results are shown as **Figure 19** .

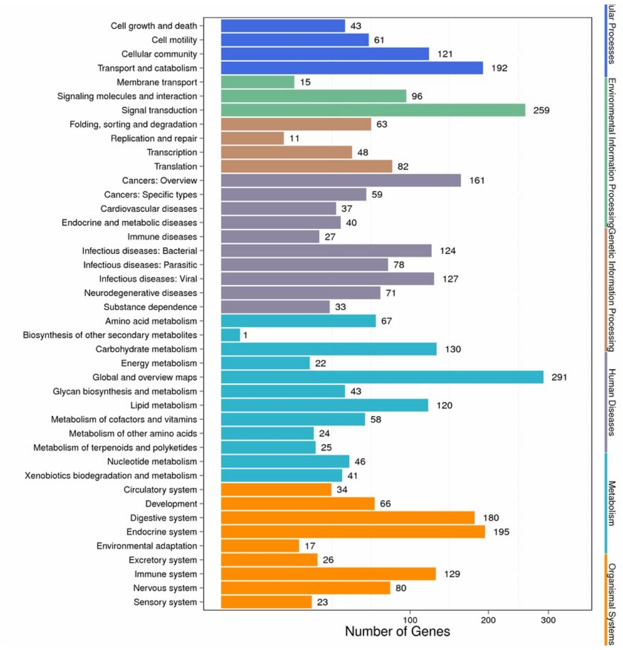

Figure 18 Pathway classification of DEGs. X axis represents number of DEG. Y axis represents pathway name.

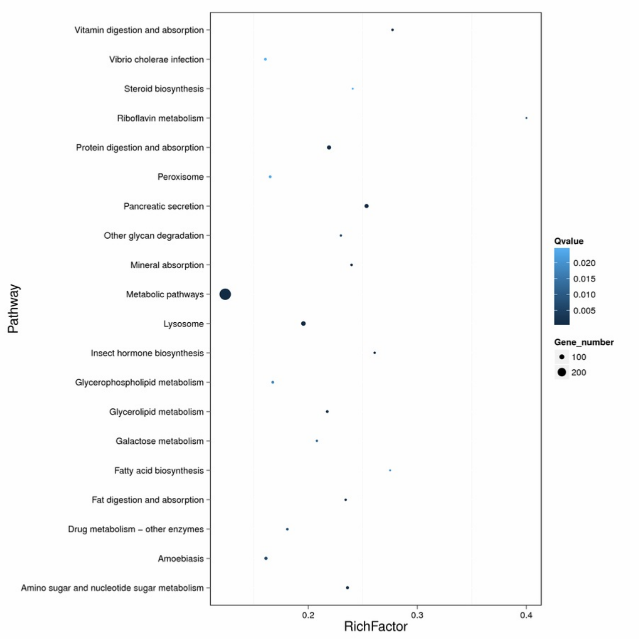

Figure 19 Pathway functional enrichment of DEGs. X axis represents enrichment factor. Y axis represents pathway name. Coloring indicate qvalue(high: white, low: blue), the lower qvalue indicate the more significant enriched. Pointsize indicate DEG number(more: big, less: small).

Methods

1 Transcriptome De novo Study Process

After extract total RNA and treated with DNase I, Oligo(dT) are used to isolate mRNA. Mixed with the fragmentation buffer, the mRNA are fragmented. Then ***cDNA*** is synthesized using the mRNA fragments as templates. Short fragments are purified and resolved with EB buffer for end reparation and single nucleotide A (adenine) addition. After that, the short fragments are connected with adapters. The suitable fragments are selected for the ***PCR*** amplification. During the QC steps, Agilent 2100 Bioanaylzer and ABI StepOnePlus Real-Time ***PCR*** System are used in quantification and qualification of the sample library. Then the library is sequenced using Illumina HiSeq 4000 or other sequencer when necessary.

After sequencing, we get raw reads. Firstly, we filter low-quality, adaptor-polluted and high content of unknown base(N) reads to get clean reads. And then perform the de novo assembly with clean reads to get the Unigenes. After that, ***SSR*** detection, Unigene expression analysis, Heterozygous ***SNP*** detection, and Unigene functional annotation are performed. Then with the functional annotation and expression results, we can detect Differentially Expression Gene(***DEG***) and perform further functional enrichment analysis between samples(two samples at least).Schematic overview of the process is shown as **Figure 1** .

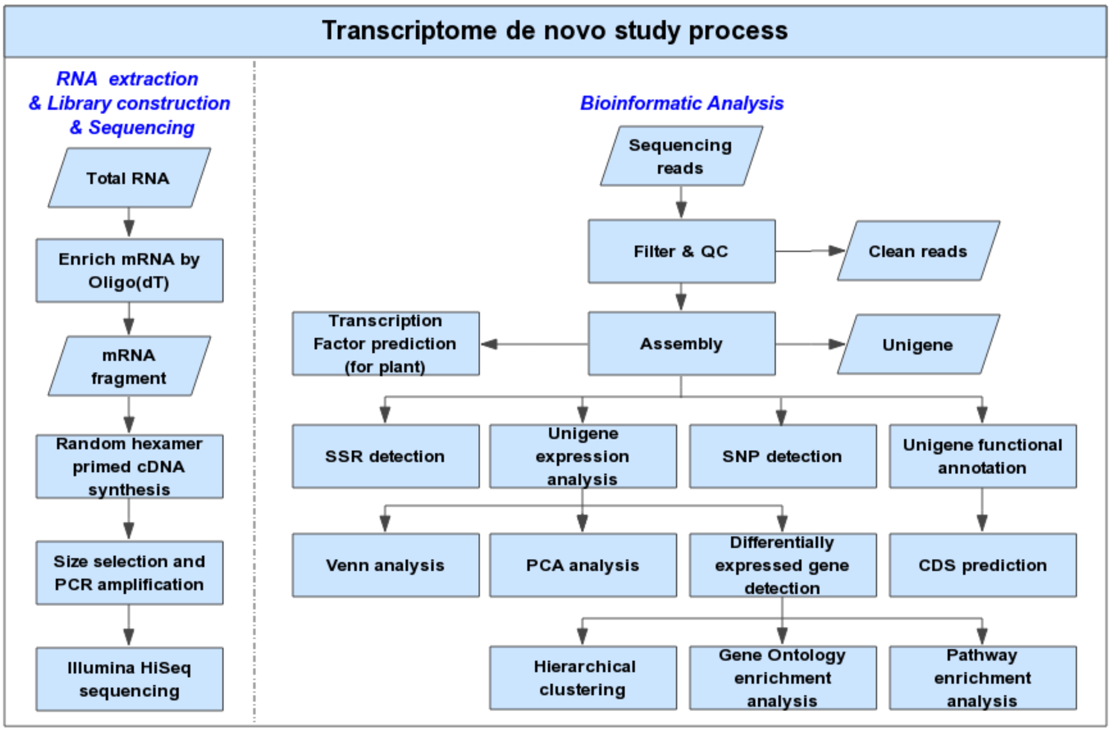

Figure 1 Transcriptome de novo study process. Schematic overview of the study process.

2 Sequencing Reads Filtering

We define raw reads as reads which containing low-quality, adaptor-polluted and high content of unknown base(N) reads additionally, these noise reads should be removed before downstream analyses. We use internal software to filter reads, fllowed as:

1) Remove reads with adaptors;

2) Remove reads in which unknown bases(N) are more than 5%;

3) Remove low quality reads (we define the low quality read as the percentage of base which quality is lesser than 10 is greater than 20% in a read).

After filtering, the remaining reads are called "Clean Reads" and stored in FASTQ [8] format (see FASTQ Format in help page).

3 De novo Assembly

We use Trinity to perform de novo assembly with clean reads that ***PCR*** duplication removed(in order to improve the efficiency), then use Tgicl to cluster transcripts to Unigenes. Trinity combines three independent software modules: Inchworm, Chrysalis, and Butterfly, applied sequentially to process large volumes of reads. Trinity partitions the sequence data into many individual de Bruijn graphs, each representing the transcriptional complexity at at a given gene or locus, and then processes each graph independently to extract full-length splicing isoforms and to tease apart transcripts derived from paralogous genes. Briefly, the process works like below:

Inchworm: Assembles the reads into the unique sequences of transcripts, often generating full-length transcripts for a dominant isoform, but then reports just the unique portions of alternatively spliced transcripts.

Chrysalis: Clusters the Inchworm Contigs into clusters and constructs complete de Bruijn graphs for each cluster. Each cluster represents the full transcriptonal complexity for a given gene (or sets of genes that share sequences in common). Chrysalis then partitions the full read set among these disjoint graphs.

Butterfly: Then processes the individual graphs in parallel, tracing the paths that reads and pairs of reads take within the graph, ultimately reporting full-length transcripts for alternatively spliced isoforms, and teasing apart transcripts that corresponds to paralogous genes.

The result sequences of Trinity is called transcripts. Then perform gene family clustering with Tgicl to get final Unigenes (if more than one sample, we would exexecute Tgicl again with each sample's Unigene to get final Unigene for downstream analyses), the Unigenes will be divided to two class, one are clusters, which the prefix is CL with the cluster id behind it(In one cluster, there are several Unigenes which similarity between them is more than 70%), the other one are singletons, which the prefix is Unigene. Schematic overview of the process is shown as **Figure 2** .

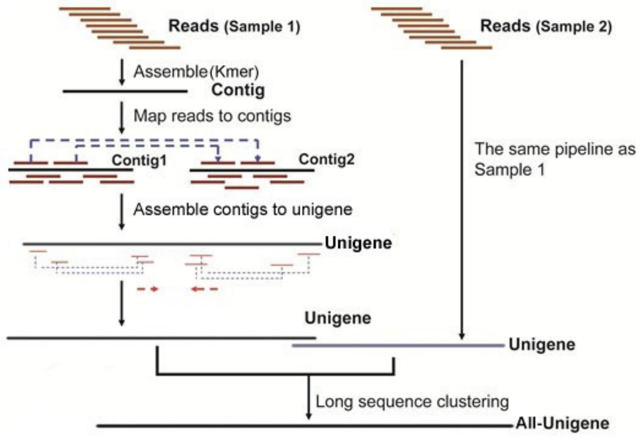

Figure 2 Assembly process. Schematic overview of the assembly process.

Software information:

Trinity:
version: v2.0.6
parameters: --min_contig_length 150 --CPU 8 --min_kmer_cov 3 --min_glue 3 --bfly_opts '-V 5 --edge-thr=0.1 --stderr'
Tgicl:
version: v2.0.6
parameters: -l 40 -c 10 -v 25 -O '-repeat_stringency 0.95 -minmatch 35 -minscore 35'

4 Unigene Functional Annotation

NT, NR, GO, COG, KEGG, SwissProt and InterPro are functional databases(for more details, please find the offical website below). We use Blast [9] align Unigenes to NT, NR, COG, KEGG and SwissProt to get the annotation, use Blast2GO [10] with NR annotation to get the GO annotation, and use InterProScan5 [11] to get the InterPro annotation. Software information:

Blast:
version: v2.2.23
parameters: default
website: http://blast.ncbi.nlm.nih.gov/Blast.cgi
Blast2GO:
version: v2.5.0
parameters: default
website: https://www.blast2go.com
InterProScan5:
version: v5.11-51.0
parameters: default
website: https://code.google.com/p/interproscan/wiki/Introduction

Database information:

NT:
description: nucleotide sequence database, with entries from all traditional divisions of GenBank, EMBL, and DDBJ excluding bulk divisions (gss, sts, pat, est, and htg divisions. wgs entries are also excluded. Not non-redundant.
website: ftp://ftp.ncbi.nlm.nih.gov/blast/db
NR:
description: non-redundant protein squence database with entries from GenPept, Swissprot, PIR, PDF, PDB and NCBI RefSeq
website: ftp://ftp.ncbi.nlm.nih.gov/blast/db
GO:
description: The ***Gene Ontology*** (GO) project is a major bioinformatics initiative to develop a computational representation of our evolving knowledge of how genes encode biological functions at the molecular, cellular and tissue system levels. Biological systems are so complex that we need to rely on computers to represent this knowledge.
website: http://geneontology.org

COG:

description: Cluster of Orthologous Groups of proteins, phylogenetic classification of proteins encoded in complete genomes.

website: http://www.ncbi.nlm.nih.gov/COG

KEGG:

description: KEGG (Kyoto Encyclopedia of Genes and Genomes) is a collection of databases dealing with genomes, biological pathways, diseases, drugs, and chemical substances. KEGG is utilized for bioinformatics research and education, including data analysis in genomics, metagenomics, metabolomics and other omics studies, modeling and simulation in systems biology, and translational research in drug development.

website: http://www.genome.jp/kegg

SwissProt:

description: UniProtKB/Swiss-Prot is the manually annotated and reviewed section of the UniProt Knowledgebase (UniProtKB).

It is a high quality annotated and non-redundant protein sequence database, which brings together experimental results, computed features and scientific conclusions.

website: http://ftp.ebi.ac.uk/pub/databases/swissprot

InterPro:

description: InterPro is a resource that provides functional analysis of protein sequences by classifying them into families and predicting the presence of domains and important sites.

website: http://www.ebi.ac.uk/interpro

5 Unigene CDS Prediction

With functional annotation, we select the segment of Unigene that best mapped to functional databases in a priority order of NR,SwissProt,KEGG,COG as its ***CDS*** , and display from 5' to 3' in FASTA fromat. Unigenes that can't be aligned to any database mentioned above are predicted by ESTScan [3] with Blast-predicted ***CDS*** as model. Software information:

Blast:

version: v2.2.23

parameters: default

website: http://blast.ncbi.nlm.nih.gov/Blast.cgi

ESTScan:

version: v3.0.2

parameters: default

website: http://sourceforge.net/projects/estscan

6 Unigene SSR Detection

We use MISA [12] to find ***SSR*** in Unigenes, then design primer for each ***SSR*** with Primer3 [13]. Software information:

MISA:

version: v1.0

parameters: 1-12,2-6,3-5,4-5,5-4,6-4 100 150

website: http://pgrc.ipk-gatersleben.de/misa

Primer3:

version: v2.2.2

parameters: default

website: http://bioinfo.ut.ee/primer3

7 SNP Detection

We mapped all clean reads to Unigenes using HISAT [14], then call ***SNP*** with GATK [4]. After filter out the unreliable sites, we get the final ***SNP*** in VCF format. Software information:

HISAT:

version: v0.1.6-beta

parameters: --phred64 --sensitive --no-discordant --no-mixed -I 1 -X 1000

website: http://ccb.jhu.edu/software/hisat/index.shtml

GATK:

version: v3.4-0

parameters(call ***SNP***): -allowPotentiallyMisencodedQuals -stand_call_conf 20.0 -stand_emit_conf 20.0

parameters(filter ***SNP***): -window 35 -cluster 3 -filterName FS -filter "FS > 30.0" -filterName QD -filter "QD < 2.0"

website: https://www.broadinstitute.org/gatk

8 Unigene Expression

we mapped clean reads to Unigenes using ***Bowtie2*** [15], and then calculate gene expression level with ***RSEM*** [16].Software information:

Bowtie2 **:**
version: v2.2.5
parameters: -q --phred64 --sensitive --dpad 0 --gbar 99999999 --mp 1,1 --np 1 --score-min L,0,-0.1 -I 1 -X 1000 --no-mixed --no-discordant -p 1 -k 200
website: http://bowtie-bio.sourceforge.net/ ***Bowtie2*** /index.shtml
RSEM **:**
version: v1.2.12
parameters: default
website: http://deweylab.biostat.wisc.edu/ ***RSEM***

9 DEG Detection

We detect DEGs with PossionDis as requested. PossionDis is based on the poisson distribution, peformed as described at Audic S, et al. [17]Software information:

PossionDis:
parameters: Fold Change >= 2.00 and ***FDR*** <= 0.001

10 Gene Ontology Analysis of DEG

With the GO annotation result, we classify DEGs according to offical classification, and we also perfrom GO functional enrichment using phyper, a function of R. The pvalue calculating formula in hypergeometric test is:

$$P = 1 - \sum_{i=0}^{m-1} \frac{\binom{M}{i}\binom{N-M}{n-i}}{\binom{N}{n}}$$

See wiki for details https://en.wikipedia.org/wiki/Hypergeometric_distribution.

Then we calculate false discovery rate(***FDR***) for each pvalue, in general, the terms which ***FDR*** not larger than 0.001 are defined as significant enriched.

11 Pathway Analysis of DEG

With the KEGG annotation result, we classify DEGs according to offical classification, and we also perform pathway functional enrichment using phyper, a function of R. The pvalue calculating formula in hypergeometric test is:

$$P = 1 - \sum_{i=0}^{m-1} \frac{\binom{M}{i}\binom{N-M}{n-i}}{\binom{N}{n}}$$

See wiki for details https://en.wikipedia.org/wiki/Hypergeometric_distribution.

Then we calculate false discovery rate(***FDR***) for each pvalue, in general, the terms which ***FDR*** not larger than 0.001 are defined as significant enriched.

Help

1 FASTQ Format

The original image data is transferred into sequence data via ***base calling*** , which is defined as raw data or raw reads and saved as FASTQ file. Those FASTQ files are the original data provided for users, including detailed read sequences and the read quality information. In each FASTQ file, every read is described by four lines, listed as follows:

```
@A80GVTABXX:4:1:2587:1979#ACAGTGAT/1
NTTTGATATGTGTGAGGACGTCTGCAGCGTCACCTTTATCGGCCATGGT
+
BMMTKZXUUUddddddddddddddddddddddddddddadddddd^WYYU
```

The first and third lines are sequences names generated by the sequence analyzer; the second line is sequence; the fourth line is ***sequencing quality*** value, in which each letter corresponds to the base in line 2; the base quality is equal to ASCII value of the character in line 4 minus 64(we call the quality system is Phred+64), e.g. the ASCII value of c is 99, then its base quality value is 35. Starting from the Illumina GA Pipeline v1.5, the range of base quality values is from 2 to 41. **Table 1** demonstrates the relationship between ***sequencing error*** rate and the ***sequencing quality*** value. Specifically, if the ***sequencing error*** rate is denoted as E and base quality value is denoted as Q, the relationship is as following formula:

$$SQ = -10 \times (\log \frac{E}{1-E})/(\log 10)$$

$$E = \frac{Y}{1+Y}$$

$$Y = \frac{SQ}{e^{-10 \times log10}}$$

Table 1 Relationship between sequencing error rate and sequencing quality value (Download)

Sequencing Error Rate(%)	Sequencing Quality Value	Character
1.00	20	T
0.10	30	^
0.01	40	h

More detaild information about FASTQ format can be got in website http://en.wikipedia.org/wiki/FASTQ_format.

Note: The quality system of Illumina HiSeq 2000(or 2500) is Phred+64, and the quality system of Illumina HiSeq 4000 is Phred+33. For the reads sequencing by Illumina HiSeq 4000, in considering of the compatibility of softwares used in our study, we will convert the quality system from Phred+33 to Phred+64 for both raw data and clean data.

2 Annotation list format

The annotation list format performed by Blast(i.e., NT, NR, COG, KEGG and SwissPro) is described as **Table 2** , and the annotation list format performed by InterProScan5(i.e., InterPro) is described as **Table 3** , and the annotation list format of GO is described as **Table 4** , and the file format of overall summarized table is described as **Table 5** .

Table 2 Format description of Blast annotation list. (Download)

Field	Description
Query_id	Unigene ID
Subject_id	Subject Seq-id (ID of the database hit)
Identity	Percentage of identical matches
Align_length	Alignment length
Miss_match	Number of mismatches
Gap	Number of gap openings
Query_start	Start of alignment in Unigene
Query_end	End of alignment in Unigene
Subject_start	Start of alignment in subject (database hit)
Subject_end	End of alignment in subject (database hit)
E_value	Expectation value (E-value)
Score	Bit score
Subject_annotation	Description of subject (database hit)

Table 3 Format description of InterProScan5 annotation list. (Download)

Field	Description
Query_id	Unigene ID
Subject_id	Subject Seq-id (ID of the database hit)
Subject_DB	Database ID
Query_start	Start of alignment in Unigene
Query_end	End of alignment in Unigene
E_value	Expectation value (E-value)
Subject_annotation	Description of subject (database hit)

Table 4 Format description of GO annotation list. (Download)

Field	Description
Unigene	Unigene ID
GO ID	Gene Ontology accession ID

Table 5 Format description of overall summarized annotation list. (Download)

Field	Description
Unigene	Unigene ID
Nr	NR annotation information, format: Subject ID / Evalue / description of subject
Nt	NT annotation information, format: Subject ID / Evalue / description of subject
Swissprot	SwissProt annotation information, format: Subject ID / Evalue / description of subject
KEGG	KEGG annotation information, format: Subject ID / Evalue / description of subject
COG	COG annotation information, format: Subject ID / Evalue / description of subject
Interpro	InterPro annotation information, format: Subject ID / Evalue / description of subject
GO	Gene Ontology annotation information, format: Category ID : GO ID // GO description;

3 What is TF

In molecular biology and genetics, a transcription factor (sometimes called a sequence-specific DNA-binding factor) is a protein that binds to specific DNA sequences, thereby controlling the rate of transcription of genetic information from DNA to messenger RNA. Transcription factors perform this function alone or with other proteins in a complex, by promoting (as an activator), or blocking (as a repressor) the recruitment of RNA polymerase (the enzyme that performs the transcription of genetic information from DNA to RNA) to specific genes. See wiki for detail https://en.wikipedia.org/wiki/Transcription_factor.

4 Gene expression list format

Gene expression result of each sample is stored in tab-seperated text file Files/BGI_result/5.Quantify/GeneExpression/**.gene.fpkm.xls*(* presents sample name) with the format description in **Table 6** .

Table 6 Format description of gene expression result list. (Download)

Field	Description
gene_id	gene ID number
transcript_id(s)	trascript list of gene, seperated by comma
length	length of gene after model regulation
expected_count	support reads number to this gene after model regulation
FPKM	FPKM value of this gene

5 DEG list format

The result of differentially expressed genes for each control-treatment pairwise is stored in tab-seperated text file Files/BGI_result/5.Quantify/DifferentExpressedGene/**.GeneDiffExpFilter.xls*(* presents pairwise name) with the format description in **Table 7** .

Table 7 Format description of DEGs screening result file. (Download)

Field	Description
Unigene	Unigene ID
Length	Unigene length
Sample1-Expression	Unigene expression of control sample(s)
Sample2-Expression	Unigene expression of treat sample(s)
log2FoldChange(Sample2/Sample1)	log2 transformed fold change between control and treat samples
Pvalue	Statistic of pvalue(PossionDis or DEseq2 method used)
FDR	Statistic of false discovery rate(PossinoDis method used)
Padj	Statistic of adjusted pvalue(DEseq2 method used)
PPEE	Statistic of posterior probability of being equivalent expression(EBseq method used)
Probability	Statistic of probability of being DEG(NOIseq method used)
Up/Down-Regulation(Sample2/Sample1)	Flags indicate up-regulated DEG(Up) or down-regulated DEG(Down) or non-DEG(*)

6 MA plot

The MA plot is a plot of the distribution of the red/green intensity ratio ('M') plotted by the average intensity ('A'). M and A are defined by the following equations:

$$M = \log_2(R/G) = \log_2(R) - \log_2(G)$$
$$A = \frac{1}{2}\log_2(RG) = \frac{1}{2}(\log_2(R) + \log_2(G))$$

See wiki for detail https://en.wikipedia.org/wiki/MA_plot.

7 Volcano plot

The Volcano plot is a type of scatter-plot that is used to quickly identify changes in large datasets, It plots significance versus fold-change on the y- and x-axes, respectively. See wiki for detail https://en.wikipedia.org/wiki/Volcano_plot_(statistics).

8 Cluster list format

The format of cluster list is described as **Table 8** .

Table 8 Format description of DEGs clustering list. (Download)

Field	Description
Unigene	Unigene ID
A-VS-B	log2FoldChange of A-VS-B
C-VS-D	log2FoldChange of C-VS-D
...	...

9 VCF format

Variant Call Format (VCF) is a flexible and extendable format for variation data such as single nucleotide variants, insertions/deletions, copy number variants and structural variants. See details at UCSC website http://genome.ucsc.edu/FAQ/FAQformat.html#format10.1

10 How to read DEG GO enrichment analysis result

Make sure that the computer has installed java and use IE brower to open *GOView.html*. The left navigation includes three types of GO terms for each control-treatment pairwise (C: cellular component, P: biological process, F: molecular function). Click one of them, the enriched GO terms result will be listed as **Figure 3** .

Gene Ontology term	Cluster frequency	Genome frequency of use	Corrected P-value	Expression Profile
BLOC complex (view genes)	2 out of 82 genes, 2.4%	8 out of 16090 genes, 0.0%	0.03943	View Result
cytosol (view genes)	2 out of 82 genes, 2.4%	15 out of 16090 genes, 0.1%	0.14450	View Result
cytosolic part (view genes)	2 out of 82 genes, 2.4%	15 out of 16090 genes, 0.1%	0.14450	View Result
intracellular part (view genes)	67 out of 82 genes, 81.7%	11513 out of 16090 genes, 71.6%	1	View Result

Figure 3 Significantly enriched GO terms in DEGs. Column 1 is GO term name. Column 2 is the ratio of DEGs enriched to this GO term. Column 3 is the ratio of genes enriched to this GO term in background database. Column 4 is Corrected P-value which indicates the degree of enrichment and the smaller Corrected P-value, the more significantly DEGs enriched to this GO term. The result list has been sorted by Corrected P-value. Column 5 is clustering of foldchange value for these enriched DEGs using the tools cluster [5] [6] and javaTreeView [7].

Click the term name 'BLOC complex' in **Figure 3** , you can go to http://amigo.geneontology.org/amigo for more information when the computer is Internet-connected. Click 'view genes' in **Figure 3** , you can get gene IDs that enriched to this GO term as **Figure 4** .

BLOC complex	63915, 100526837
cytosol	63915, 100526837

Figure 4 Gene ID list related to GO terms. There are two DEGs enriched to the term 'BLOC complex': 63915, 100526837.

11 How to read DEG pathway enrichment analysis result

Open html report for pathway enrichment result and the enriched KEGG pathways will be listed as **Figure 5** .

1. sample3-VS-sample4

#	Pathway	DEGs with pathway annotation (1432)	All genes with pathway annotation (17252)	Pvalue	Qvalue	Pathway ID
1	Pathways in cancer	81 (5.66%)	531 (3.08%)	5.562454e-08	1.074132e-05	ko05200
2	Focal adhesion	74 (5.17%)	475 (2.75%)	8.877128e-08	1.074132e-05	ko04510
3	Leukocyte transendothelial migration	46 (3.21%)	280 (1.62%)	5.86161e-06	3.950743e-04	ko04670
4	Rheumatoid arthritis	25 (1.75%)	115 (0.67%)	6.530153e-06	3.950743e-04	ko05323
5	Malaria	19 (1.33%)	76 (0.44%)	1.00329e-05	4.855924e-04	ko05144

Figure 5 Pathway enrichment analysis of DEGs. Column 1 is ordinal number. Column 2 is pathway name. Column 3 is the ratio of DEGs enriched to this pathway. Column 4 is the ratio of genes enriched to this pathway in background database. Pvalue and Qvalue are both values that indicate the degree of enrichment and Qvalue is corrected Pvalue. The smaller they are, the more significantly DEGs enriched to this pathway. The result list has been sorted by Qvalue. The last column pathway ID corresponding to pathway name.

Click pathway name 'Leukocyte transendothelial migration' in **Figure 5** , you can get gene IDs that enriched to it as **Figure 6** .

3	Leukocyte transendothelial migration	146850, 654463, 5909, 4318, 1364, 402415, 3383, 2888, 100528016, 5175, 9404, 149461, 285590, 5880, 50507, 79778, 58494, 8572, 8481, 6525, 5603, 90799, 55691, 100506649, 29970, 4739, 6876, 55679, 5010, 9076, 9411, 26509, 9758, 10398, 8727, 7412, 7070, 6387, 8502, 7430, 7414, 71, 60, 4771, 80014, 51306
4	Rheumatoid arthritis	2921, 6364, 6374, 3576, 3553, 4319, 2920, 2919, 3552, 4314, 2353, 4312, 3589, 100288077, 3383, 7099, 7422, 1514, 7040, 533, 7042, 6387, 284, 5157, 6347

Figure 6 Gene ID list related to pathway. There are 46 DEGs enriched to the pathway 'Leukocyte transendothelial migration'.

Furtherly, detecting the most significant pathways, the enrichment analysis of ***DEG*** pathway significance, allows us to see detailed pathway information in KEGG database. For example, clicking the hyperlink on 'Leukocyte transendothelial migration' in **Figure 6** will get detailed information as shown in **Figure 7** .

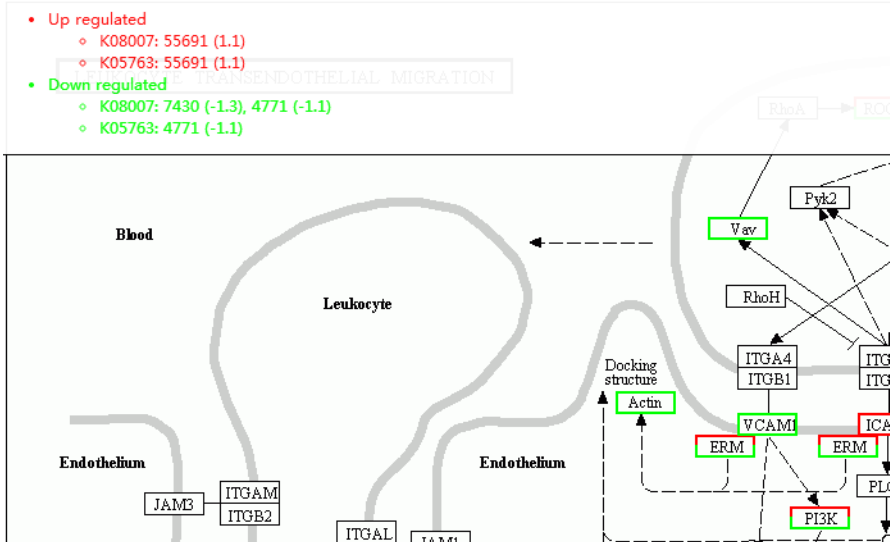

Figure 7 An example of KEGG pathway of 'Leukocyte transendothelial migration'. Up-regulated genes are marked with red borders and down-regulated genes with green borders. Non-change genes are marked with black borders. When mouse hover on border with red or green, the related DEGs appear on the top left. Clicking gene name in the figure, the page will redirect to KEGG website if the computer is Internet-connected.

References

[1] Grabherr MG, et al.(2011).Full-length transcriptome assembly from RNA-Seq data without a reference genome. Nat Biotechnol. 2011 May 15;29(7):644-52.
[2] Pertea G, et al.(2002).TIGR Gene Indices clustering tools (TGICL): a software system for fast clustering of large EST datasets.Bioinformatics (2003) 19 (5): 651-652.
[3] Iseli C, et al.(1999).ESTScan: a program for detecting, evaluating, and reconstructing potential coding regions in EST sequences.Proc Int Conf Intell Syst Mol Biol. 1999:138-48.
[4] McKenna A, et al.(2010).The Genome Analysis Toolkit: a MapReduce framework for analyzing next-generation DNA sequencing data.Genome Res. 2010 Sep;20(9):1297-303.
[5] Eisen, M. B., et al. (2001). Cluster analysis and display of genome-wide expression patterns. Proc Natl Acad Sci USA, (1998)95(25): 14863-8. 2001.29: 1165-1188.
[6] M. J. L. de Hoon, et al. (2004). Open Source Clustering Software.Bioinformatics, 20(9): 1453-1454.
[7] Saldanha, A. J. (2004). Java Treeview--extensible visualization of microarray data. Bioinformatics, 20(17): 3246-8.
[8] Cock P., et al.(2010). The Sanger FASTQ file format for sequences with quality scores, and the Solexa/Illumina FASTQ variants. Nucleic Acids Research, 38(6): 1767-1771.
[9] Altschul SF, et al.(1990).Basic local alignment search tool.J Mol Biol. 1990 Oct 5;215(3):403-10.
[10] Conesa A, et al.(2005).Blast2GO: a universal tool for annotation, visualization and analysis in functional genomics research.Bioinformatics. 2005 Sep 15;21(18):3674-6.

[11] Quevillon E, et al.(2005).InterProScan: protein domains identifier.Nucleic Acids Res. 2005 Jul 1;33(Web Server issue):W116-20.
[12] Thiel T, et al.(2003).Exploiting EST databases for the development and characterization of gene-derived SSR-markers in barley (Hordeum vulgare L.).Theor Appl Genet. 2003 Feb;106(3):411-22.
[13] Untergrasser A, et al.(2012).Primer3 - new capabilities and interfaces.Nucl. Acids Res. (2012) 40 (15): e115.
[14] Kim D, et al.(2015).HISAT: a fast spliced aligner with low memory requirements. Nature Methods 2015.
[15] Langmead B, et al.(2012).Fast gapped-read alignment with Bowtie 2. Nature Methods. 2012, 9:357-359.
[16] Li B, et al.(2011).RSEM: accurate transcript quantification from RNA-Seq data with or without a reference genome.BMC Bioinformatics. 2011 Aug 4;12:323.
[17] Audic S, et al.(1997).The significance of digital gene expression profiles.Genome Res. 1997 Oct;7(10):986-95.

2015 Copyright BGI All Rights Reserved 粤ICP备 12059600 Technical Support E-mail:info@bgitechsolutions.com Website: www.bgitechsolutions.com
